# Death of Jules Guerin

**Published:** 1886-03-20

**Authors:** 


DEATH OF JULES GUERIN.—The oldest member of the French Academy of Medicine, died recently at the age of eighty-five. He was an original and eccentric character, and it is said opposed with great bitterness the modern germ theory, especially in the case of cholera, and was very severe on Pasteur's method of innoculating against hydrophobia.

ELECTRICITY.—Wait & Bartlett, manufacturing Electricians, have an advertisement in this issue of our journal, which our readers are requested to examine. Every thing in the line of Electro-therapeutics should be studied by the practitioner. A doctor who desires to keep pace with the advances in the profession cannot afford to ignore the great advantages of electricity in the treatment of many otherwise intractable forms of disease.

